# Supplementary material for: Are carbon emissions trading and green financial instruments synergistic? -Comprehensive quantitative research based on content analysis
Source: PLoS One. 2024 Mar 7;19(3):e0298601. doi: 10.1371/journal.pone.0298601 (PMC10919601; doi:10.1371/journal.pone.0298601)
Supplement: S1 Data — (ZIP) [file pone.0298601.s001.zip › Data/地级市Z_F工作报告.zip!/╣π╢1⁄2/╘╞╕í╩╨/└┤╫╘╣1⁄2╓┌║┼í╛┬φ┐╦╩2╛▌═°í┐_2010.docx]

各位代表：

我代表市人民政府向大会作政府工作报告，请予审议，并请政协各位委员和其他列席人员提出意见。

2009年工作回顾

2009年是我市全面推进建设广东富庶文明大西关的开局之年，也是我市全力应对国际金融危机冲击，抢抓机遇、化危为机、克难奋进、加快发展的一年。一年来，在省委、省政府和市委的正确领导下，在市人大及其常委会和市政协的监督支持下，市政府坚持以科学发展观统领经济社会发展全局，坚决贯彻执行中央“保增长、保民生、保稳定”和省“三促进一保持”、“双转移”等重大决策部署，大力推进“两区四城一纽带”建设，较好地完成了市四届人大五次会议提出的经济社会发展主要预期目标任务。

——经济增长目标基本实现。全市完成生产总值347.43亿元，比2008年（下同）增长10.5%，其中一、二、三产业增加值分别增长6%、10.8%和13.5%；人均生产总值14397元，增长9%；单位GDP能耗下降3.5%；规模以上工业增加值95.43亿元，增长10.5%；农业总产值146.15亿元，增长6.5%；社会消费品零售总额119.52亿元，增长18.9%；全社会固定资产投资总额240.19亿元，增长45.1%；地方财政一般预算收入18.78亿元，增长12.9%；城镇居民人均可支配收入13211元，增长7.1 %；农村居民人均纯收入6128元，增长11.6%；居民消费价格总指数下降0.8%；人口自然增长率5.49‰；城镇登记失业率2.9%；外贸进出口总额9.51亿美元，下降17.7%，比年初收窄16个百分点；出口总额6.16亿美元，下降13%，比年初收窄17.2个百分点；实际利用外资0.71亿美元，增长3.1%。

——重点工程（项目）建设目标基本完成。2009年开展的50项重点工程（项目）建设是我市保增长的重要引擎。经过一年的努力，50项重点工程（项目）共完成投资116.61亿元,完成年计划的107.9%。其中，要求年内完成的28项，已有 25项基本建成或完成年度投资计划；要求年内动工的16项，已有15项动工；要求重点推进的3项和要求立项的3项工程（项目）进展也逐步加快。

——交通基础设施建设实现新突破。全市共投入交通基础设施建设资金50.41亿元,同比增长84.7%。“五三二一”工程[广梧、云岑、江罗、阳罗、汕湛五条高速公路；云浮新港六都四围塘码头、都杨通用码头（煤码头）、温氏都杨码头三个码头；南广高铁、罗岑铁路两条铁路；一段西江航道整治]建设提速，进展顺利，当年完成投资41.29亿元，完成年计划的133%。其中，广梧高速（河口至平台段）已累计完成投资的86.15%，今年“五一”前全线通车；云岑高速（双凤至双东段）全面动工；阳罗、江罗和汕湛高速（都杨至天堂段）前期工作提速推进，其中阳罗、江罗高速将于今年动工。云浮新港（六都四围塘码头）顺利开港运营，都杨通用码头（煤码头）已动工建设，温氏都杨码头前期工作加快。南广高铁（云浮段）完成年计划投资的161.4%；罗岑铁路全面复工建设。西江航道（云浮段）整治工程顺利进行。与此同时，国道324线罗定段路面大修、河杨公路扩建首期工程已完成。

——人居环境建设呈现新亮点。建设“健康、生态、幸福”的宜居城市示范市工作获得社会各界广泛支持和参与。建成宜居城市一期（南山森林公园及周边市民活动设施改造）工程，市民满意率达96%；二期“上山”、“进城”工程快速推进，已完成自行车休闲道和部分慢行系统工程建设。成功创建省级卫生城市，启动创建国家卫生城市工作。完成星岩路和城区供水及应急供水改造工程，城区管道燃气首期工程已建成供气。新建生态文明村600条。云安县成功创建省级林业生态县，实现我市五个县（市、区）均为生态县的目标。新兴金水台温泉、六祖故里旅游度假区被评定为国家4A级旅游景区。按五星级标准建设的城区金凯莱酒店、罗定国际大酒店已基本建成。房地产市场蓬勃发展，商品房施工面积、销售面积和收入同比分别增长52.3%、107.7%和120.9%。全面开展《云浮市城市总体规划》编制，围绕做大做优中心城区，积极构建“一江三组团”城市格局。云城、云安同城化工作顺利推进，都杨新城区产业发展和基础设施建设加快。

——招商引资和双转移工作呈现新气象。通过构建融资平台、市县共建共享、财政贴息贷款等措施，全力推进园区加快建设。佛山（云浮）产业转移工业园成功评为省第三批示范性产业转移工业园，竞获省扶持资金5亿元，利用佛山市共建资金5亿元（已到账1亿元），并向金融机构融资15亿元和向国家发改委申报10亿元国债项目。各园区基础设施建设和项目入园工作加快。其中，佛山（云浮）产业转移工业园都杨片区已累计投入9.75亿元，入园项目34个，计划投资12亿元，另有意向项目96个，计划总投资150亿元；佛山顺德（新兴新成）产业转移工业园已投入2.6亿元，入园项目27个，计划总投资11.03亿元；罗定双东环保工业园已投入1亿元，入园项目5个，计划总投资25.88亿元，郁南县以“一园两区”的模式建设双东环保工业园大湾分园，实现共建共享；云浮循环经济工业园新入园项目15个，计划总投资25.64亿元。市教育园区已完成征地、规划和部分项目融资等工作，其中市中专新校区（含市体育中心）、市技工学校实训基地、实验小学和云安县凤凰高级中学等项目累计投入资金4.09亿元。经贸招商工作取得较大成效。成立了招商引资专门机构，聘任了100名招商顾问，制订了招商引资鼓励政策，有效调动了全社会招商引资的积极性；成功举办了云浮（广州）投资环境推介会、云浮（佛山）投资环境推介会和第六届石展会等经贸洽谈活动，与省直部门签署了信息化建设、共建硫化工基地等7个领域的合作协议。全年经贸活动共签约项目206个，总投资146.63亿元。劳动力转移就业培训扎实推进。全市共新增转移农村劳动力5.12万人，组织技能等级培训3.52万人，培训后转移就业3.14万人，转移就业率达89%。

——特色产业集群发展实现新提升。制定了《云浮市特色工业发展规划（2009-2015年）》等9个工业发展规划，确定了打造五大百亿产业集群的发展目标，规划到2013年，把石材、不锈钢制品产业，到2015年，把水泥、硫化工、电力产业分别打造成为年产值超百亿元的产业集群。大力实施企业成长工程，全市共新增规模以上企业100家、新登记企业827家。组建省级产学研创新平台和示范基地6个，新增省级以上企业技术中心3家，其中温氏集团被认定为国家级企业技术中心。郁南电池产业集群获省批准为我市第三个省级产业集群升级示范区。节能降耗工作取得较大进展。全市累计淘汰落后水泥产能347万吨，规模以上工业增加值能耗同比下降5.3%。重大工业项目进展顺利，投资27.9亿元的云浮发电厂C厂已完成大部分主体工程，将于今年上半年试产；总投资13亿元的青洲水泥、12亿元的广东贝铝等一批项目先后动工建设；总投资50亿元的华润西江发电厂首期项目，省发展改革委已同意开展前期工作，并已上报国家发展改革委；总投资15亿元的华润集团（罗定）水泥项目已成功签约；云浮核电站项目正抓紧申报进入国家和省的核电发展规划。

——农村改革发展迈出新步伐。制定了《云浮市农村改革发展试验区规划纲要（2009-2020年）》，省已批准我市为全省农村改革发展试验区。农村居民养老保险试点和集体林权制度改革全面启动，打造国家级畜牧业产业化示范中心、农村饮水安全、水库除险加固、城乡防灾减灾、生态防护林、现代农业园区、沼气池、医疗卫生等工作继续推进并取得新成效。新兴被授予“全国农产品加工基地”，郁南10万亩沙糖桔、罗定10万亩稻谷生产基地被评为全国绿色食品原料标准化基地，云安被授予“中国优质沙糖桔之乡”。全市农业产业化组织发展到240个，增加45个；实现销售收入207亿元，增长6.2%；带动合作农户户均增收7023元。温氏集团实现销售收入170亿元，增长7.6%。开发补充耕地4.92万亩，粮食播种面积178.9万亩，总产量66.48万吨，粮食工作考评各项指标完成省下达任务。

——财税金融工作取得新成绩。全市共组织地税收入15.29亿元,增长18%；组织国税收入13.08亿元，下降 14.8%，但降幅逐月、逐季收窄；组织财政总收入51.16亿元，增长19.7%。坚持公共财政理念，重点保障了社会事业、民生工程、宜居城市建设等支出。全市共完成财政总支出50.53亿元，增长17.5%，其中一般预算支出48.54亿元，增长17.4%；落实中央和省厉行节约要求，全市共节约财政资金8476万元。金融机构存贷款持续增长，本外币存款余额405.35亿元，比年初增长20.9%，贷款余额214.93亿元，增长55.1%。政银企合作取得重大成果，我市分别与四大商业银行和国开行签订了授信总额共556.8亿元的融资协议，至去年底已落实贷款104.99亿元；各金融机构与“五大”百亿产业集群对接融资项目1403个，计划贷款金额72.1亿元。郁南县农村金融改革发展综合试点进展顺利。广东温氏集团发行的16亿元企业债券成功注册，首期9亿元已上市发行；广东大华农动物保健品股份有限公司已完成上市申报，广东凌丰集团股份有限公司已进入上市辅导期。

——社会民生发展有新提高。全市参加新农合人数200.74万人，覆盖率99.5%；住院补偿13.2万人次，补偿金额2.79亿元，分别增长17%和56.1%。参加城镇医保19.6万人，比2008年增加1.1万人，新型农村养老保险在新兴县成功试点，养老保险待遇不断提高。动工建设市人民医院新院，市中医院新综合大楼投入使用，提前完成20间乡镇薄弱卫生院改造和200间村卫生站规范化建设任务。积极做好应对甲流等重大疫情防控和病人救治工作，全市没出现暴发重大疫情和食物中毒事件。健全城乡低保救助、五保供养、优抚对象抚恤补助自然增长机制，全市共发放低保金6034.38万元、五保金3136.27万元、优待抚恤金3525.58万元，分别增长36.5%、22.3%和8.43%。“规划到户、责任到人”扶贫开发工作全面铺开，全市87个贫困村和3.4万贫困户全部落实帮扶责任单位和帮扶责任人。行政村（居委会）客车通达率达90.3%，新建“万村千乡市场工程”农家店115家，各级财政安排3596万元补贴家电、汽车、摩托车下乡以及家电以旧换新，农村消费活跃，农村居民生产生活条件进一步改善。建设廉租住房508套，缓解了部分城镇低收入家庭的住房困难问题。申报省级以上科技项目210项，其中省部产学研合作项目61项，增长52.5%，获省科技进步奖项目5项。落实义务教育阶段免费和高等、中职教育助学政策，享受国家义务教育免费金额1.06亿元，受惠学生39.05万人次；落实了中小学教师工资福利“两相当”和教师“代转公”工作；高中阶段毛入学率68.8%，比上年提高13个百分点，高考上本科人数比上年增加725人；市特殊教育学校建成开学。打造禅宗六祖文化、南江文化和石艺文化品牌取得新成绩，一批文物和非物质文化遗产项目被列入国家、省级保护名录，桔乡农民合唱团获首届全国农民合唱大会“岐江杯”奖，音乐剧《六祖惠能》获全省“五个一工程”奖，“广东禅文化创意产业园”获省批准建设；市人民广场被评为“全国特色文化广场”，罗定市、新兴县被复评为全国先进文化县，罗定博物馆被评为国家级博物馆；文化市场繁荣稳定，扫黄打非工作取得新成绩。继续保持低生育水平，我市连续两年被省政府授予“广东省人口与计划生育先进单位”，新兴县被授予2009年度全国计划生育优质服务先进单位。环境质量继续改善，全市二氧化硫排放量比上年下降26%，市区空气优良率97.8%，同比提高5个百分点。认真落实国家和省“安全生产年”工作要求，较好地完成了省下达安全生产各项考核控制指标任务。平安和谐工程成效明显，群众对社会治安的满意率达95.1%，越级上访大幅减少，我市综治信访维稳中心建设经验得到上级的充分肯定。

——政府管理上新水平。编制了《云浮市资源环境城乡区域统筹发展规划》和《云浮市改革发展规划纲要（2009—2020年)》。我市分别被省政府批准为“三网融合”（电信网、广播电视网、互联网）和“三规合一”（国民经济和社会发展规划、城乡总体规划、土地利用总体规划）试点市。至去年底，已完成市区3000户“三网融合”试点，启动罗定试点，市民对试点工作满意率达99.4%。探索建立“镇级公共财政城乡均等化机制”、“干部考核评价机制”、“共建共享机制”等相关工作积极推进。加强政府自身建设，增强公务员队伍大局意识、发展意识、机遇意识、责任意识和实干意识。认真落实市人大及其常委会各项决议、决定并向其报告工作，加强与人民政协的联系并向其通报情况，密切与工会、共青团、妇联等人民团体的联系，广泛听取人大代表、政协委员和各民主党派、工商联、无党派人士的意见建议，全年共办理人大建议73件、政协提案191件。建立健全了领导干部问责、电子监察、巡视督办、监督通报、工作报告、考评奖惩等制度，把全市重点工作和50项重点工程(项目)逐一分解给市领导班子成员和相关部门,明确责任,推动落实。开展第四轮行政审批制度改革，基本完成市级政府机构改革。认真落实廉政建设制度，促进权力阳光运行，各级政府和公务员队伍勤政、廉政意识进一步增强。

此外，物价、统计、人事人才、编制、经济发展研究、知识产权、民族宗教、档案、外事侨务、港澳事务、台务、人民防空、地方志、消防、保密、气象、侨联、文联、社科联、妇儿、工商联、红十字会、贸促会、广播电视、新闻出版以及驻市外机构等工作也取得新成绩。

各位代表！过去一年政府工作取得的成绩，来之不易。这是在省委、省政府和市委正确领导下，全市上下抢抓机遇、开拓进取、共克时艰、奋力拼搏的结果。在此，我代表市人民政府，向全市人民、驻云浮部队官兵和关心支持云浮发展的港澳台同胞、海外侨胞及国际友人表示衷心的感谢并致以崇高的敬意！

回顾一年的工作，我们深深体会到，实践科学发展观，对于云浮来说，当前的核心就是努力实现又好又快的跨越发展：一要狠抓项目建设不放松，持续数年保持投资的高速增长，为实现跨越发展奠定基础。二要狠抓招商引资和推进“双转移”不放松，通过加快引进新的项目，不断壮大支柱产业，增强经济发展后劲。三要狠抓机制创新不放松，积极探索推动科学发展、跨越发展的新思路、新举措。四要狠抓效能建设不放松，增强干部队伍的干劲，倡导实干精神，在干字上下功夫，努力提高政府的施政水平和服务效率。

同时，我们清醒地看到，我市经济社会发展还存在一些亟待解决的突出问题。主要是：经济总量小，发展不充分，发展载体不完善，支柱产业不强；招商引资成效不明显；城镇化水平较低，社会事业发展滞后；一些地方思想解放程度不够高，加快发展的责任感不够强，加快发展的工作作风不够实，落实加快发展的办法不够多。等等。对于这些工作薄弱环节，市政府将高度重视，采取有效措施切实加以解决。

2010年工作安排

2010年是实施“十一五”规划的最后一年，是我市乘势而上，实现科学发展、跨越发展的关键一年，做好今年工作意义重大。在新的一年里，我市机遇与挑战并存，希望大于挑战。国际经济逐步复苏，国家继续实施积极的财政政策和适度宽松的货币政策，我省深入推进“双转移”战略，新出台的《关于促进粤北山区跨越发展的指导意见》给予了山区市很多倾斜政策，并把我市定位为“全省农村改革发展试验区、循环经济和人居环境建设示范市、广东富庶文明大西关”，再次明确把我市确定为重点发展地区，大力支持我市20多个重大项目加快建设。今年，我们确定了投资总额608.42亿元、年内完成投资156.96亿元的60个重点工程（项目）。这是我市加快发展的重要抓手。我们必须抓住机遇，全力推进经济社会科学发展、跨越发展。

2010年政府工作的总体要求是：以邓小平理论和“三个代表”重要思想为指导，深入贯彻落实科学发展观和十一届全国人大三次会议、省委十届六次全会、粤西地区（云浮）现场会、粤北山区工作会议、市委四届七次全会精神，积极落实《珠江三角洲地区改革发展规划纲要》，全面实施《云浮市改革发展规划纲要（2009—2020年）》，大力推进“三促进一保持”，以美好环境建设为载体，积极探索以人为本的发展机制，坚持好中求快、又好又快，加快转变经济发展方式，加快融入珠三角，打造珠三角最具活力的经济延伸区，着力加强以保障和改善民生为重点的社会建设，全面完成“十一五”规划各项目标任务，全面加快“全省农村改革发展试验区、循环经济和人居环境建设示范市、广东富庶文明大西关”建设步伐。

2010年经济社会发展主要预期目标是：生产总值增长12.5%，人均生产总值增长11.5%，单位生产总值能耗下降3.2%，化学需氧量控制在2.2万吨以内，二氧化硫排放量控制在4万吨以内，研发经费支出占GDP比重1.5%，服务业增加值占GDP比重33.6%，全社会固定资产投资总额增长30%，进出口总额增长10%，实际利用外资增长10%，社会消费品零售总额增长18%，地方财政一般预算收入增长13%，居民消费价格总指数涨幅控制在3%以内，城镇登记失业率控制在3%以内，人口自然增长率控制在7‰以内，城镇居民人均可支配收入增长10%，农村居民人均纯收入增长9.5%。

为实现上述目标,今年市政府将紧紧围绕“积极推进双转移，努力实现跨越发展”这条主线，抓好以下十个方面的工作：

一、加快转变经济发展方式，推动产业集聚发展

实施特色工业发展规划，加快传统特色产业转型升级，培育发展新兴产业，突出抓好投资总额104.67亿元的21个重点项目建设，年内完成投资56.54亿元，推动产业集聚发展，壮大经济总量。

着力打造“五大”百亿产业集群。石材产业着力围绕产业升级推进国家级石材检测中心、云浮石材博览中心、云安新型石材基地等项目建设，加强政策扶持，引导石材产业上规模、创品牌。不锈钢产业着力在巩固发展国际市场的同时，大力开拓国内市场，加快筹建不锈钢制品国家级企业技术研发机构，办好不锈钢产业创业园。水泥产业着力加快中材罗定水泥、青洲水泥一线、中材亨达400万吨粉磨站等在建项目进度，推动华润集团（罗定）水泥和中材亨达三线等项目动工。硫化工产业以云硫集团为龙头，着力拉长产业链，加快惠沄钛白、立宇矿业电解锰等项目建设，并通过以商引商，大力发展下游产品，加快壮大产业规模，培育中国硫化工重要产业基地，创中国驰名商标。电力产业着力在确保云浮电厂C厂上半年并网发电，争取华润西江发电厂年内获得国家发改委同意开展前期工作文件的同时，积极发展清洁能源，推进风电、核电进程，年内完成罗定风电和新兴风电项目建议书，并向省发改委申报争取获得项目核准文件；争取中广核核电项目列入省相关规划并上报国家，开展编制可研阶段各项工作；中电投核电项目通过初可评审，并争取列入省相关规划。

着力培育新兴产业集群。组织编制全市健康产业发展规划，整合传统产业资源，培育新的产业集群。大力培育机械装备、新型材料、“三网融合”应用、生物制药等新兴低碳产业。

着力扶持中小企业做大做强。继续抓好工业企业成长工程，实行市、县（市、区）领导班子成员挂点联系企业制度，切实解决企业的实际困难，为企业发展创造良好的政务环境，促进中小企业做大做强。年内新增规模以上工业企业100家，新培育私营企业200家。支持企业开展科技创新，重点扶持成长性好、创新能力强、有自主品牌的企业实现转型升级。支持广东大华农动物保健品股份有限公司、广东凌丰集团股份有限公司等企业上市，实现我市企业在资本市场融资零的突破。

着力发展循环经济。积极实施资源节约、综合利用等循环经济工程，推动传统产业形成资源循环利用的产业链条。通过技术创新、管理创新、资源整合，切实有效地推进石材、硫化工、水泥等传统产业节能减排、转型升级，实现可持续发展。

二、加快交通基础设施建设，全力融入珠三角

加快总投资391.66亿元的15个重点项目建设，年内完成投资63.09亿元，加快构建广东大西关交通网络，做好与珠三角地区、粤西地区以及大西南地区的交通对接，凸显云浮的竞争优势。

全力推进“五三二一”工程。力争广梧高速（河口至平台段）今年“五一”前全线通车；云岑高速确保年内完成投资14亿元，力争2011年通罗定城区；江罗、阳罗高速争取年内动工；汕湛高速（都杨至天堂段）完成工可专家评审，争取启动土地预审、环评等前期工作。年内动工建设温氏都杨码头，建成都杨通用码头（煤码头）主体工程。确保南广高铁（云浮段）、罗岑铁路年内分别完成投资22亿元和6亿元；基本完成西江航道（云浮段）整治。

全力推进国省道、县乡公路改造建设。年内完成国道324线云城云安段、省道352线南江口至素龙段、省道276线新兴过境公路段大修工程，以及河杨公路扩建工程二期（都杨工业园至沿江公路段）工程，开工建设河口街市政综合开发工程，规划兴建新城快速干线、都杨新区站前路。同时，继续抓好县乡公路的改造建设。

全力推进后备项目立项攻关。继续抢抓省大力支持山区加快基础设施建设的机遇，规划立项一批重点交通基础设施项目，争取年内完成肇云轻轨的线路规划和工可等前期工作，努力实现交通基础设施与珠三角的全面对接，为全力融入珠三角创造条件。

三、加快推进双转移，实现招商引资新突破

狠抓招商引资。围绕年内实现全市招商引资150亿元的工作目标，创新招商引资方式，营造政府主导、园区带动、全社会参与招商引资的良好氛围，重点突破引进大企业、大项目尤其是大型国有企业、上市公司、台资企业和珠三角地区列入“三旧”（旧城镇、旧厂房、旧村庄）改造范围的企业到我市投资发展。落实招商引资工作责任制，市领导带头招商引资，年底开展工作责任考核。在加强对外招商的同时，开展对企业的“直通车”服务，引导扶持现有企业增资扩产、拓展关联产业，推动企业上规模、上水平。

狠抓园区建设。落实园区建设工作责任制，推动“四大园区”建设新突破，重点推进征地、招商引资和园区基础设施建设，并力促一批企业投产。佛山（云浮）产业转移工业园（都杨片）年内投入基础设施建设资金8亿元，引进企业50个（其中投产30个），引资额突破50亿元；佛山顺德（新兴新成）产业转移工业园要加快二期项目开发，完善基础设施建设，强化主导产业，提高招商引资和项目建设成效，年内引资30亿元；云浮循环经济工业园要充分整合资源，完善基础设施，推动关联企业建立科学的循环经济产业链，年内引资30亿元，努力争创省级示范园区；罗定双东环保工业园以及郁南大湾分园要进一步完善发展规划，加快基础设施建设，瞄准电子、电镀、五金、化工、纺织等产业招商，年内引资50亿元（其中双东园区引资30亿元，大湾分园引资20亿元）。与此同时，以佛山（云浮）产业转移工业园为基础，整合相关资源，申报设立“广东省云浮高新技术产业开发区”。

狠抓劳动力培训转移。积极实施全民技能提升储备计划，完善“校企合作”、“工学结合”、“技能进村”、“结对培训”等培训模式，推动农村劳动力转移有序化、技能化和规模化。年内完成农村劳动力技能培训3万人，新增转移农村劳动力5万人。全面加快市教育园区建设，年内完成市中专新校区一期（含市体育中心）、市技工学校实训基地、实验小学和云安凤凰中学等项目主体工程建设。

四、加快人居环境示范市建设，打造“值得作为故乡的城市”

全面实施《云浮市美好环境与和谐社会建设共同缔造行动纲要》，努力把云浮打造成为“宜居的珠三角后花园”、“宜业的珠三角产业延伸区”和“值得作为故乡的城市”。

做大中心城区。加快云城、云安同城化和都杨新区建设，完善连接“一江三组团”交通网络，年底完成沿江公路（六都至都杨段）建设，构起“一江三组团”城区框架，为打造与地级市相适应的中等城市奠定基础。加快各县城、中心镇基础设施建设和服务业发展，增强辐射带动能力。加快推进碧桂园、臻汇园、东方时代广场、恒晖苑、三河洲三期等房地产项目和市人民医院新院、城区供水管网等公共服务项目，以及南山河防洪工程等公共基础设施建设。组织实施“三旧”改造,启动几个示范改造项目并取得明显成效。全面开展创建国家卫生城市工作。

做优生态环境。全面实施《云浮市以宜居城乡建设为目标推进绿色生态工程实施方案》。加强生态公益林建设，大力开展城市绿化、美化，实施林业生态文明“万村绿”工程。规划建设全市生态文化旅游慢行绿道系统，实现与珠三角绿道网相衔接。抓好宜居城市二期工程，加快“上山”、“进城”项目建设，分期推进城区慢行系统建设，为市民提供健康、生态、幸福的人居环境。推进宜居城镇、宜居村庄、宜居社区建设，积极探索农村生活垃圾和污水处理的有效途径，进一步改善村容村貌。

五、加快现代服务业发展，培育新的经济增长点

全力加快投资总额75.23亿元的11个重点服务业项目建设，年内完成投资20.76亿元，努力提升全市服务业整体水平，力争服务业增加值占GDP比重达33.6%，成为全市新的经济增长点。

大力发展特色旅游业。全面实施特色旅游发展规划，加大旅游资源整合和宣传推介力度，加强旅游合作，大力发展六祖文化、生态观光、休闲度假、乡村民俗等特色旅游。完善旅游景区基础设施和服务设施建设，加快新兴龙山禅泉、郁南新永光度假村等上规模、上档次旅游度假项目建设。积极参加“全国旅游服务质量提升年”和“华人华侨旅游年”主题活动，办好云浮市2010年旅游文化节。

大力发展物流等现代服务业。编制全市现代物流产业发展规划，加强与大型物流企业合作，启动云浮新港（六都四围塘码头）物流园区建设。推进供销合作社改革发展，继续实施“万村千乡市场工程”，组织家电、汽车、农机、摩托车、建材下乡等活动，扩大农村消费。发展生产服务业和电子商务、连锁经营、物流配送等现代营销业，积极发展保险、中介等新型服务业，健全现代服务产业体系。

大力开拓市场，扩大开放。加快海关、出入境检验检疫和边防检查等口岸联检单位的设施建设，落实鼓励出口的政策措施，优化口岸通关环境，提高通关效率，降低进出口成本，促进对外经贸业务健康发展。深入研究国内外市场的新变化，积极开拓新的市场，培育新的出口企业，帮助符合条件的民营企业申报出口经营权，壮大出口规模。积极组团参加省组织的广东名优产品北京展销会、第六届泛珠洽谈会、第六届山洽会、中博会等经贸招商活动，办好第七届石展会，以此推介我市投资环境，加强经贸合作，提高知名度。

六、加快现代生态农业发展，促进农民持续增收

加大农业投入。加大基本农田保护工作力度，严格保护耕地，继续利用园地、山坡地开发补充耕地，确保全市粮食播种面积稳定在180万亩以上、产量在66万吨以上。全面落实惠农政策，抓好病险水库加固、机电排灌、现代标准农田和农业综合开发、沼气池等工程建设，年内完成金银河、罗光、共成、向阳和云霄等五座中型水库除险加固，新建农村沼气池2000座。试点推进“村村通自来水工程”，打造一批新农村示范点。认真执行省以县为单位考核公共基础设施建设的“插红旗”制度，加快农村水、电、路等设施建设，改善农业和农村发展条件。

发展现代生态农业。围绕打造“珠三角健康食品生产基地”目标，充分利用我市生态环境优势和农业产业化基础，重点发展优质畜禽、优质大米、特色果蔬等健康食品以及食品加工等高附加值产业。推广标准化、无公害农业生产，加强农产品商标注册和地理标志保护工作，培育农业品牌。大力发展农资连锁经营、农产品现代购销、农业技术、农业信息等农村服务业，推进郁南县两广农产品加工物流园区建设，加快建设一批特色鲜明、集散功能较强的农产品批发交易市场。完善农产品质量检测、动植物疫病检测防控等设施建设。全力推进“打造国家级畜牧业产业化示范中心”工作，发展壮大农业龙头企业，提升全市农业产业化经营水平，增强农民持续增收能力。

七、加快改革创新，在先行先试上取得新突破

全面组织实施《云浮市改革发展规划纲要（2009—2020年）》，先行先试，努力在体制机制上创造优势，为全省乃至全国提供新鲜经验，形成推动全市科学发展、跨越发展的强大动力。

创新管理机制。深入实施“三规合一”机制，完善相关规划，积极争取省和国家的重视和支持，加快建立以《云浮市资源环境城乡区域统筹发展规划》为核心的“一套规划、统一编制、分头实施”的规划体系，构建“一个平台、统一标准、分类管理”的规划管理体系，实现国民经济和社会发展规划、城乡总体规划以及土地利用总体规划在编制、实施与管理上的统一。加快推进“三网融合”扩面工作，年内网络覆盖扩大至整个城区，并逐步向县、镇、村延伸。落实共建共享机制，努力调动各级、各部门发展积极性。改革考核措施，量化考核指标，充分发挥考核机制对促进科学发展的导向作用。创新扶贫开发机制，把“规划到户、责任到人”工作与建立农村社会救济、农业保险等农村社会保障体系结合起来，通盘考虑，积极探索集扶贫开发、社会救济、社会保险为一体的农村社会保障体系。创新城乡统筹机制，打破城乡二元结构，改革户籍管理制度，推进以“居住证”为核心的“一证通”制度，推动农民向城镇转移，实现城乡统筹发展。同时，编制好“十二五”规划，引领云浮实现科学发展、跨越发展。

创建农村改革发展试验区。改革农村土地管理制度，开展土地流转市场建设试点，加快建立农村土地承包经营权流转市场；推进农村金融改革，建立引导信贷资金和社会资金投向农村的激励机制，畅通农村投融资渠道，切实解决“三农”融资难问题；积极发展涉农保险业务，开发涉及种养的新险种，降低农业产业风险；推进农村社会保障改革，加快建立新型农村社会养老保险制度；改革农村基层干部激励机制，逐步落实村干部报酬正常增长、养老保险、离职补偿等激励保障措施；推进集体林权制度改革，抓好林业产权交易中心建设，加强林权动态管理，促进林业经营权规范有序流转，年内基本完成林改主体改革。

八、加快社会各项事业发展，促进基本公共服务均等化

加快教育、科技事业发展。健全和完善义务教育经费保障机制，抓好义务教育学校布局调整和规范化学校建设，优化教育资源配置，促进城乡教育均衡发展。加快普及高中阶段教育，今年力争毛入学率达80%；积极发展民办教育、幼儿教育和特殊教育。完善中小学教师工资福利待遇“两相当”制度和绩效工资改革工作。大力推动科技创新，以创新能力、创新体系、创新环境为重点，加大科技项目建设投入，深入开展产学研科技合作，为经济社会发展提供有力的科技保障。

加快文化、卫生、体育、人口计生等事业发展。加快推进文化强市建设进程，深入推进文化体制改革，完善公共文化服务体系，全面推进“农家书屋”、农村电影放映工程建设，大力促进城乡公共文化均等化。加快规划建设广东禅文化创意产业园和云浮石艺（创意）产业园，培育新型文化产业。加强文物和非物质文化保护，进一步挖掘禅宗六祖文化、南江文化、石艺文化等地方特色文化精华，打造云浮城市品牌文化，提升文化软实力。推进医疗卫生体制改革，加强公共卫生服务和基层医疗卫生服务体系建设，加大公共卫生重点项目投入，大力发展中医药事业。全面贯彻落实国家《全民健身条例》，广泛开展全民健身运动，推动竞技体育上台阶、上水平。稳定低生育水平，建立依法管理、政策推动、村民自治、综合治理新机制，全面完成人口和计划生育目标管理任务。组织实施第六次全国人口普查工作。加强环境综合治理，确保完成减排目标。加强人民防空、民兵预备役建设和拥军优属、优抚安置工作，进一步巩固和发展全省双拥模范城成果。

保障广大群众切身利益。加强以保障和改善民生为重点的社会建设。推进统一规范的人力资源市场建设，构建城乡一体化公共就业服务平台，切实抓好大中专毕业生、下岗失业人员等就业困难群体的就业工作。落实城乡最低生活保障、农村五保、社会救助和城乡居民医疗保障等措施，提高保障水平，年内把农村人均年纯收入低于1500元的困难家庭全部纳入低保范围，农村五保供养标准要达到当地人均收入的60%以上。加强残疾人事业、老龄事业和慈善事业发展。推进民生水利工程和保障性住房建设，年内解决25万农村群众的饮水安全问题，新建廉租住房351套。

努力维护社会稳定。推进镇街综治信访维稳中心建设，坚持市领导信访接待日制度和领导包案制度，及时解决群众反映的热点难点问题，有效预防和妥善处置各类群体性事件。推进“平安云浮”建设，积极创新社会管理，不断完善社会治安防控体系。大力推动和开展法治县（市、区）活动。严格落实安全生产责任制，加强应急管理体系建设，提高处置突发重大公共事件和自然灾害的能力。

九、加强财税金融工作，提高保障支撑能力

积极培育财源。坚持依法治税，加强税源监控与征收，严厉打击偷、漏、逃、抗税行为，抓好国有资产、行政事业单位资产、城市公共资产和国土资源经营收益征缴以及其他非税收入管理工作，千方百计增加财政收入。制定完善有利于支柱产业做大做强的财政激励政策，营造有利于企业成长的政策氛围。强化预算执行刚性，优化财政支出结构，加大对民生和公共事业的投入，继续严格执行厉行节约各项要求，压缩一般性开支，确保财政收支平衡。

提高金融机构存贷比。坚持政、银、企定期会商制度，切实做好“四大园区”、“五大百亿产业集群”和今年确定的60项重点工程（项目），以及“三农”项目的融资协调工作，着力提高金融机构存贷比，保持金融贷款快速增长的良好势头，提高金融对经济的保障支撑能力。

十、加强政府自身建设，提高政府执行力

加强民主法制建设。坚持和完善向人大及其常委会报告工作、向人民政协通报情况制度，自觉接受监督。认真办理人大代表议案、建议和政协提案。积极支持政协委员发挥参政议政作用。主动听取各民主党派、工商联、无党派人士的意见，支持工会、共青团、妇联、工商联等人民团体更好地发挥作用。充分利用“三网融合”平台，通过行风评议、政风行风热线、市长热线、城市论坛等形式，扩大公众参与科学决策、民主监督的渠道，推动政府工作科学化、民主化、法治化、规范化。

提高行政效能。推进县级政府机构改革，抓好事业单位分类改革。继续精简行政审批事项，减少审批环节，提高审批效率。创新服务方式，进一步完善电子政务、电子监察、网上审批和“一条龙、一站式”服务，提高办事效率。

转变机关作风。加大公务员学习培训力度，全力创建学习型机关。大力倡导抓落实的良好政风，提高行政执行力。坚决纠正有令不行、有禁不止的行为，确保政令畅通。坚决制止“乱作为”，防止出现“不作为”。加大对重点专项工作和重点工程项目的督查力度，确保项目安全、资金安全。以加强对行政权力的监督制约为重点，深入推进政府系统廉政建设，坚持勤俭办事，反对铺张浪费，努力建设一支开拓进取、团结务实、奋发创业、勤政廉政的公务员队伍。

此外，继续加强物价、统计、人事人才、编制、民族宗教、档案、外事侨务、港澳事务、台务、地方志、消防、保密、气象、工会、青年、妇儿、侨联、文联、残联、社科联、工商联、红十字会、贸促会以及驻市外机构等工作。

各位代表！时代赋予我们更大责任，人民群众寄予我们更多期待。我们坚信，只要坚定不移地贯彻落实科学发展观，坚定不移地落实市委、市人大的决策部署，坚定不移地依靠广大干部群众，就一定能够克难奋进、抓住机遇，不断开创改革发展新局面。让我们在市委的坚强领导下，坚定信心，乘势而上，开拓创新，全面完成今年的目标任务，为推动全市经济社会科学发展、跨越发展，建设广东富庶文明大西关作出新的更大的贡献！
